# Electromagnetically induced absorption in a three-resonator metasurface system

**DOI:** 10.1038/srep10737

**Published:** 2015-05-29

**Authors:** Xueqian Zhang, Ningning Xu, Kenan Qu, Zhen Tian, Ranjan Singh, Jiaguang Han, Girish S. Agarwal, Weili Zhang

**Affiliations:** 1 Center for Terahertz waves and College of Precision Instrument and Optoelectronics Engineering, Tianjin University, and the Key Laboratory of Optoelectronics Information and Technology (Ministry of Education), Tianjin 300072, China; 2 School of Electrical and Computer Engineering, Oklahoma State University, Stillwater, Oklahoma 74078, USA; 3 Department of Physics, Oklahoma State University, Stillwater, Oklahoma 74078, USA; 4 Center for Disruptive Photonic Technologies, Division of Physics and Applied Physics, School of Physical and Mathematical Sciences, Nanyang Technological University, 21 Nanyang Link, Singapore 637371, Singapore

## Abstract

Mimicking the quantum phenomena in metamaterials through coupled classical resonators has attracted enormous interest. Metamaterial analogs of electromagnetically induced transparency (EIT) enable promising applications in telecommunications, light storage, slow light and sensing. Although the EIT effect has been studied extensively in coupled metamaterial systems, excitation of electromagnetically induced absorption (EIA) through near-field coupling in these systems has only been sparsely explored. Here we present the observation of the EIA analog due to constructive interference in a vertically coupled three-resonator metamaterial system that consists of two bright and one dark resonator. The absorption resonance is one of the collective modes of the tripartite unit cell. Theoretical analysis shows that the absorption arises from a magnetic resonance induced by the near-field coupling of the three resonators within the unit cell. A classical analog of EIA opens up opportunities for designing novel photonic devices for narrow-band filtering, absorptive switching, optical modulation, and absorber applications.

As a composite matter made up of subwavelength resonators, metamaterials possess unusual and exotic electromagnetic properties that give rise to new capabilities in manipulating electromagnetic waves in a desired manner^1^,^2^,^3^,^4^,^5^,^6^,^7^. Experimental and theoretical investigations have shown that plasmonic coupling in the near-field mode plays an essential role in enabling the optical and electromagnetic properties of metamaterials^8^,^9^,^10^. In-depth understanding of the fundamental coupling mechanisms not only gives remarkable insight into the design and optimization of metamaterial building blocks with desirable functionalities, but also leads to many intriguing phenomena. Among these, the EIT analog is a result of the coupling between a bright and a dark resonator, where the destructive interference of the resonance modes delivers a sharp window of nearly perfect transmission within a broad absorption band. The EIT effect has been recently mimicked in various metamaterial approaches, including cut wires^11^,^12^,^13^, bilayer fish-scale structures^14^, split-ring resonators (SRRs)^15^,^16^,^17^,^18^,^19^ and asymmetric Fano resonators^20^. This effect drastically modifies the dispersive properties of an otherwise opaque medium, which leads to fascinating potential applications, such as slow light^11^,^15^,^16^, photonic switching^15^,^16^, loss reduction^17^ and sensing^13^,^19^.

In contrast to the destructive interference of the coupled EIT resonators, a constructive interference of different excitation pathways would lead to a new fascinating phenomenon, namely, the EIA. Instead of a pronounced transparency window, a sharp absorption resonance is induced in the EIA system. This effect has been realized in two coupled resonator systems by introducing a retardation-induced phase shift in an intermediate coupling regime^21^,^22^, manipulating their dissipative loss and coupling strength^23^, or stimulating them with different phase via oblique incidence^24^, where the absorption resonance generally emerged from the absorption envelop background.

In this article, we present experimental observation of a unique EIA effect in a three-resonator system. The proposed vertically coupled metasurface system features three types of resonance modes^25^ with a nearly identical resonance frequency but different damping rates. The design capitalizes on three coupled resonators in which the pronounced sharp absorption resonance is induced within an EIT window, splitting the original EIT resonance into two windows. In addition, we show that the EIA resonance is a result of constructive interference induced magnetic response of the near-field coupled three-resonator metasurface system.

## Results

### Sample schematic and experimental results

**Figure 1:**
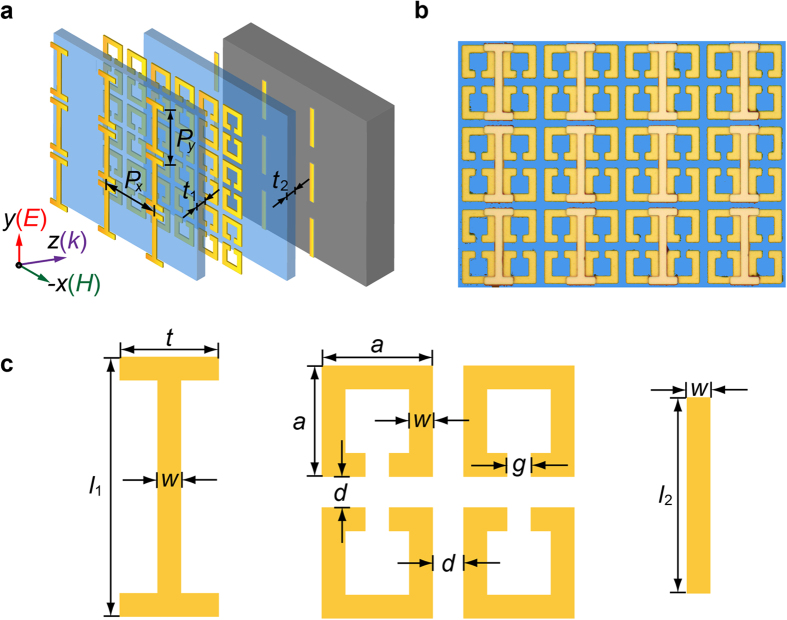
Sample schematic and microscopic image. (**a**) Schematic diagram of the three-layered EIA metasurface unit cell. (**b**) Microscopic image of the EIA sample. It was fabricated on a silicon substrate and the dielectric spacer is made from polyimide. (**c**) Schematic of the individual resonators. *P*_*x*_ = *P*_*y*_ = 120 μm, *t*_1_ = *t*_2_ = 10 μm, *l*_1_ = 110 μm, *l*_2_ = 83 μm, *t* = 42 μm, *w* = 10 μm, *a* = 47 μm, *d* = 13 μm, and *g* = 10 μm, respectively.

Figure 1a illustrates the schematic of a three-layered metasurface unit cell which is excited at normal incidence by the terahertz electromagnetic wave. The metamaterial comprises of three different types of metallic subwavelength resonators, namely, an I-shaped structure at the top layer, a four-SRR structure at the middle layer and a cut-wire structure at the bottom layer, respectively. Each adjacent metasurface layer is spaced by a 10 μm-thick polyimide, thus the overall thickness of the sample is only 20 μm (except the substrate). Figure 1b displays a microscopic image of the fabricated sample and shows a precise alignment between the three-layer resonators (see Methods). Figure 1c illustrates the design with geometrical parameters. The thickness of the polyimide spacer is much less than the terahertz resonance wavelength. Thus, the structure could be considered as an effective medium and the close proximity of the layers also ensures the near-field coupling between the resonators^26^.

**Figure 2:**
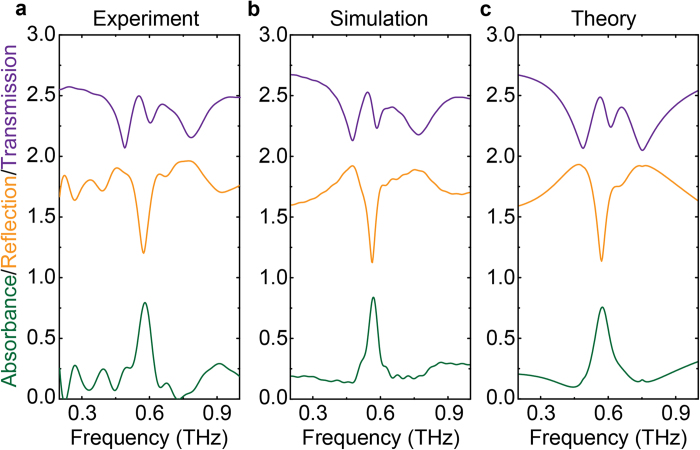
Amplitude transmission, reflection and absorbance spectra. (**a**) Experimental, (**b**) simulated and (**c**) theoretical spectra of the amplitude transmission (purple), reflection (orange) and absorbance (green) under normally incident *y*-polarized wave. In (**c**), the parameters used in the calculation are: *ω*_t_ = 2*π* × 0.61 THz, *ω*_b_ = 2*π* × 0.60 THz, *ω*_m_ = 2*π* × 0.64 THz; *γ*_t_ = 2*π* × 0.018 THz, *γ*_m_ = 2*π* × 0.013 THz, *γ*_b_ = 2*π* × 0.007 THz; *g*_t_ = 2*π* × 5.57 MHz, *g*_b_ = 2*π* × 87.54 MHz; *κ*_t-m_ = 2*π* × 0.0001 THz, *κ*_b-m_ = 2*π* × 0.13 THz; *d*_p_ = 20 μm, *d*_s_ = 625 μm; ∆*φ* = 0.22 rad. The offsets along the *y*-axis reflection and transmission are 1.0 and 2.0, respectively.

To investigate the optical properties of the EIA system, the transmission *t*(*ω*) and reflection *r*(*ω*) spectra at normal incidence were measured using a terahertz time-domain spectroscopy (THz-TDS) system^27^ (see Methods). The frequency-dependent absorbance was then calculated as *A*(*ω*) = 1 − |*t*(*ω*)|^2^ − |*r*(*ω*)|^2^. The experimentally measured |*t*(*ω*)|, |*r*(*ω*)| and *A*(*ω*) are shown in Fig. 2a. In the transmission spectrum, a dip resonance, originated from the EIA effect, was developed from a broad EIT resonance, thus also showing a double-peak EIT behavior. The EIA resonance was further validated by the corresponding sharp resonance dip in the reflection spectrum. This unique feature enables the distinct absorption characteristic in the absorbance spectrum with a relatively sharp EIA window of 70 GHz (full width at half maximum) at 0.58 THz with an intensity of 0.78. It can be seen that the central resonance frequencies of the reflection and transmission spectra at the EIA window are slightly deviated from each other. They, however, can be adjusted to overlap with each other by adjusting the geometrical parameters of the structures.

### Simulation and analysis

**Figure 3:**
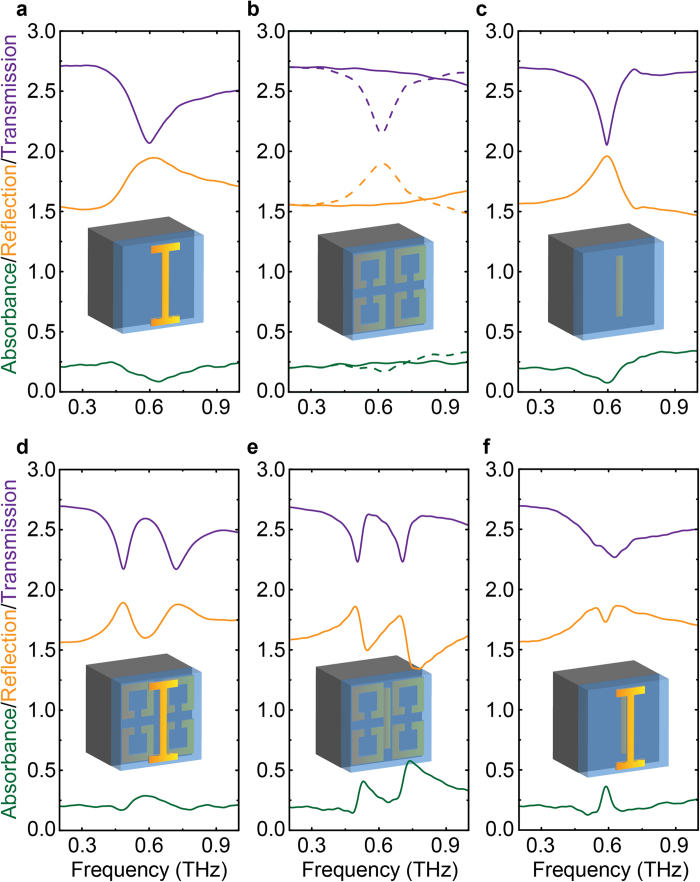
Simulation results of individual and cascaded resonators. Amplitude transmission (purple), reflection (orange) and absorbance (green) spectra of individual resonators (**a**–**c**) and three cases of two combined resonators (**d**–**f**), as indicated by the inset diagrams. The resonance frequencies of the resonators in (**a**–**c**) are 0.6, 0.6 and 0.62 THz, respectively. All the solid curves are obtained under the normally incident *y*-polarized wave, while the dash curves in (**b**) are achieved under the normally incident *x*-polarized wave.

Numerical simulations on spectral response of the EIA system was performed by using the commercial software package *CST Microwave Studio* (see Methods). The simulation results are in good agreement with the experimental measurements, as shown in Fig. 2b. To further explore the coupling effect between the resonators within a unit cell, additional simulations were carried out on the individual resonators and combinations of two of these resonators. Figure 3 presents the simulated transmission, reflection and absorbance spectra of various structures under *y*-polarized incidence (solid curves) and *x*-polarized (dashed curves, in Fig. 3b only) incidence, respectively. The incident wave polarized along the *y* direction directly excited the I-shaped and the cut-wire resonators (see Fig. 3a,c) whereas the four-SRR resonator could not be directly excited (see Fig. 3b). When the I-shaped and four-SRR resonators (Fig. 3d), or the cut-wire and four-SRR resonators (Fig. 3e) are combined together, a typical EIT type spectral response is observed with a pronounced transparency window. Therefore, the I-shaped and the cut-wire resonators act as the bright modes as they are directly excited by the incident field, while the four-SRR resonator acts as the dark mode resonator since they are excited by the near-field coupling from the bright mode resonators. Furthermore, it is interesting to see that when the two bright resonators are cascaded together (see Fig. 3f), a broader but weaker resonance occurs. However, none of the absorbance spectra in Fig. 3 displays an absorption resonance which is as strong as that in the three-resonator system, although the cases in Fig. 3e,f reveal a small absorption feature. Therefore, the remarkable EIA effect in the proposed multilayer system is indeed attributed to the vertical coupling between all the three resonators.

**Figure 4:**
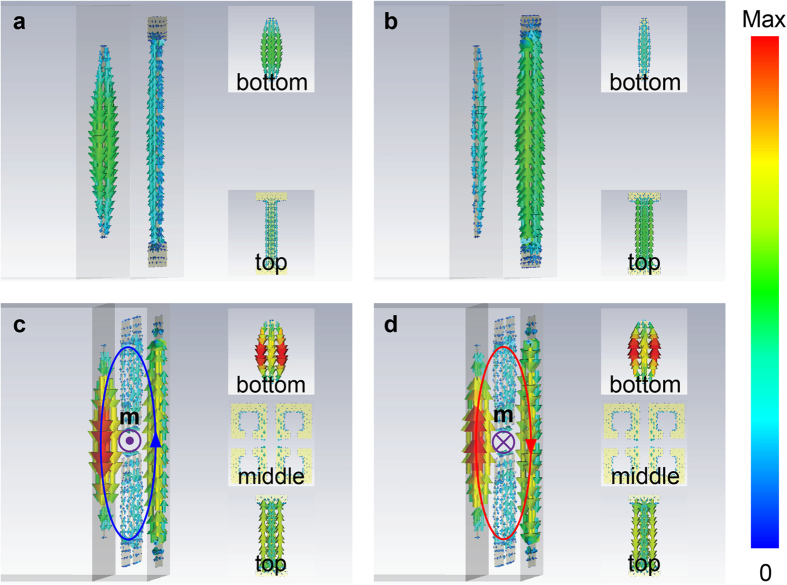
Simulated surface current distributions. (**a**,**b**) Simulated surface current distributions for the case shown in Fig. 2(f) with *φ* = 1.44π and 1.83π at resonance frequency. (**c**,**d**) Simulated surface current distributions for the three-layer case with *φ* = 1.44π and 0.44π at the peak absorption frequency. Insets: corresponding surface current distributions of each layer resonator.

In order to elucidate the physical process involved, we calculated the electromagnetic surface current distributions of selected designs (see Methods). For the bright resonators, the surface currents of the top I-shaped resonator and bottom cut-wire resonator display a ~ 0.4π phase difference at the resonance frequency. In other words, when the current in the I-shaped resonator is the strongest, the current in the cut-wire resonator nearly becomes the weakest, and vice versa, as seen in Fig. 4a,b. After adding the four-SRR resonator as the middle layer, the surface current distributions in the two bright resonators are significantly altered because of their near-field coupling with the dark SRRs as the intermediate layer. Figure 4c,d indicate that there is a phase difference of π between the I-shaped and cut-wire resonators at the peak absorption frequency. As a result, the anti-parallel currents form a magnetic dipole, as indicated by a current ring. Different from the trapped mode in the planar metamaterial^28^, the magnetic dipole direction **m** is along the *x*-axis, which is same as the incident magnetic field polarization. Therefore, it strongly traps the incident magnetic energy, thus giving rise to a strong absorption. On the other hand, the weak absorption shown in Fig. 3f is caused by the small net magnetic dipole since the phase difference of the anti-parallel currents is not exactly π/2. It can also be clearly seen that the surface currents at the top and bottom resonators are both enhanced in comparison with the case shown in Fig. 4a,b due to the constructive interference effect in the EIA system. It should be noticed that the EIA effect described here does not convert the transparency window into a dip, but rather creates an absorption resonance within the transparency window which is formed from the coupling between either one of two bright resonators and the dark resonator. Therefore, the EIA resonance here does not alter the original high transmissions at the two separated EIT window frequencies.

**Figure 5:**
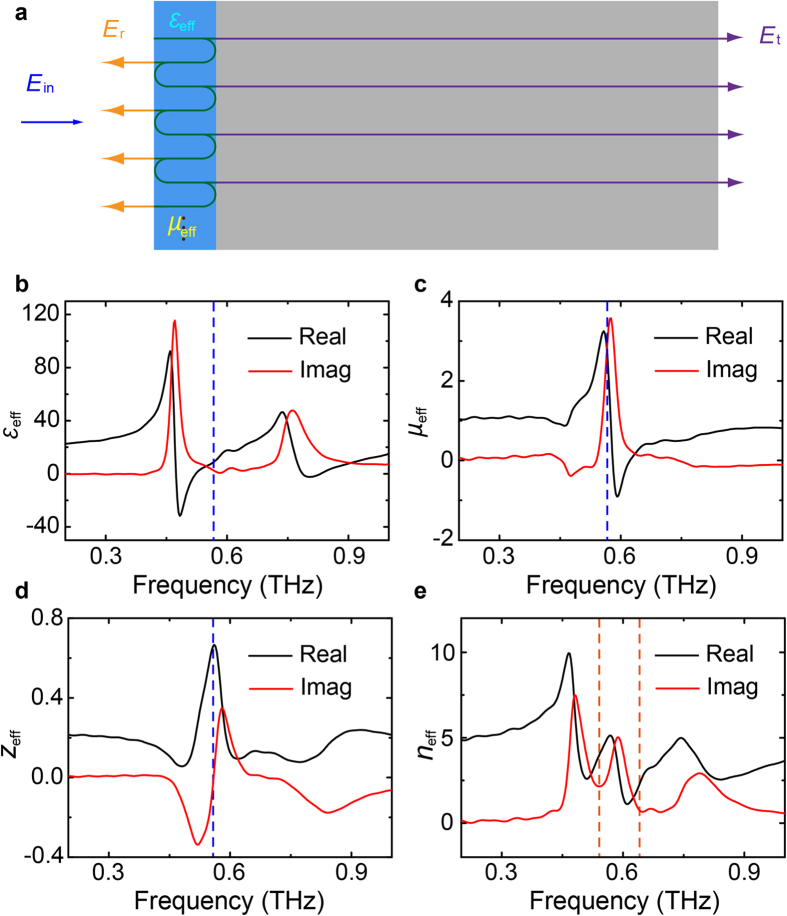
Effective permittivity and permeability. (**a**) Schematic of the retrieval model. (**b**, **c**) Retrieved effective permittivity *ε*_eff_ and permeability *μ*_eff_ of three-resonator EIA metamaterial with simulated results. (**d**, **e**) Retrieved effective impedance *z*_eff_ and refraction index *n*_eff_ with simulated results. The black and red curves represent their corresponding real and imaginary components. The blue dashed lines indicate the location of the peak absorption frequency, while the orange dashed lines indicate the location of the separated EIT-peak frequencies at 0.54 and 0.64 THz, respectively.

To confirm that the absorption phenomenon is resulted from the near-field coupling induced magnetic response, effective parameters of the EIA metamaterial are retrieved by solving the Fresnel’s equations using the transmission and reflection spectra^29^. The three-layered structure in the polyimide is treated as an effective medium, as illustrated in Fig. 5a. By using the simulated transmission and reflection results, we obtained the effective permittivity *ε*_eff_ and permeability *μ*_eff_, respectively, as shown in Fig. 5b,c. It is observed that *μ*_eff_ changes dramatically at the peak absorption frequency owning to the anti-parallel currents. The remarkable imaginary part of *μ*_eff_ also represents a strong absorption to the incident electromagnetic wave. However, the anti-parallel currents reveal a weak contribution to *ε*_eff_ since the electric response of the top and bottom resonances tend to cancel each other due to the π phase difference. This is clearly demonstrated in Fig. 5b that the other two resonances without anti-parallel currents only contribute to *ε*_eff_. Though the imaginary parts of *ε*_eff_ at these two resonances are also obvious, the absorptions are quite weak. This can be attributed to the strong mismatch in the effective impedance *z*_eff_ which results in strong reflection; whereas at the magnetic resonance, *z*_eff_ is between the impedances of air and the substrate, thus the wave can easily penetrate the effective media and be absorbed, as illustrated in Fig. 5d. Figure 5e illustrates the effective refraction index *n*_eff_, where the strong frequency-dependent dispersion feature indicates a strong slow-light property at the two separated EIT-peak frequencies.

### Theoretical model

To describe the resonance behaviors of the three resonators in the EIA system, three coupled equations (in rotating-wave approximation) were used^25^:
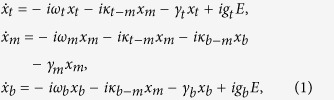
where *x*_*i*_, *ω*_*i*_ and *γ*_*i*_ with *i* ∈{t, m, b} are the amplitudes, resonance frequencies and damping rates of the top, middle and bottom resonators, respectively. *g*_t_, *g*_b_ are coupling strength of the top and bottom resonators to the external field *E*. The intermediate resonator can only be excited by the coupling from the top resonator with coupling coefficient *κ*_t-m_ and the bottom resonator with coupling coefficient *κ*_b-m_. According to equation (1), we obtain the susceptibilities of the two bright resonators *χ*_t_ and *χ*_b_, respectively. Thus the transmission and reflection coefficients at the two interfaces with bright resonators are calculated utilizing the method described in Ref. 15 by neglecting the ultrathin thickness of aluminum and taking *d*→0 limits:
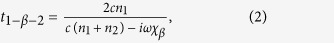

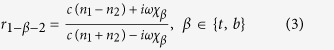
where equations (2) and (3) are obtained under a condition that the incident wave passes through metamaterial layer *β* from medium 1 with refractive index *n*_1_ to medium 2 with refractive index *n*_2_. Here *c* is the light velocity in vacuum and *ω* represents the angular frequency. With these obtained coefficients, the overall transmission and reflection coefficients of the EIA system are calculated as
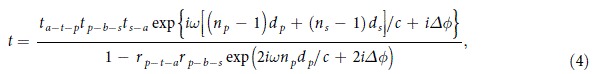

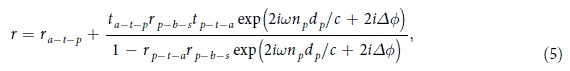
in which the multi-reflection effect in the thin polyimide layer is also considered^30^; where the subscripts {a, t, b, p, s} denote air, top resonator, bottom resonator, polyimide spacer and silicon substrate, respectively; *n* and *d* represent the refractive index and thickness; ∆*ϕ* is the phase change introduced by the dark mode. Figure 2c shows the calculated result based on the model, showing excellent agreement with the measurements and the simulations.

## Discussion

In conventional metamaterial absorbers^31^,^32^,^33^, the absorption is also found to be related to the vertically anti-parallel magnetic currents, but their transmission is extremely low in a broadband frequency range (for example, continuous metal film acts as a ground plane). The EIA effect gives out a way that such strong absorption resonance may also occur but without compromising high transmission amplitude except at the EIA window. This three-resonator design delivers a strong EIA up to 78% which is much stronger than the existing two resonator metamaterial systems. Besides, along with the EIA resonance, the original transparent window splits into a double-peak EIT-like behavior, which could be useful in developing slow-light devices with dual band transparency. From simulations, we also found that the response of the EIA system reveals strong interlayer distance dependence. As other coupled-resonator system, the transmission and reflection spectra are very sensitive to the position of the SRRs in the polyimide layer which determines its coupling strength to the top and the bottom structures. A very small variation in the vertical or horizontal position of the SRRs leads to an apparent modification in the spectral response. This property could be used in developing metamaterial rulers at terahertz frequencies and plasmonic rulers at infrared and optical frequencies^34^. Furthermore, by introducing semiconductor or superconductor into our system, the response can be effectively modulated from EIT to EIA by external excitations.

In conclusion, we demonstrate the quantum phenomenon of EIA in a vertically coupled tri-layer classical metasurface. The scheme relies on a three-coupled-resonator unit cell, where a strong magnetic response gives rise to a strong absorbance. The near-field coupling mechanism presented here would allow the design of EIA analogs across a broad spectrum of the electromagnetic waves. This composite metamaterial design also opens up a new route to classical analogs of quantum effects leading to photonic devices with promising applications in light switching, absorption, and sensing.

## Methods

### Sample fabrication

The three-resonator EIA metamaterial was fabricated on a 625 μm silicon substrate (*ε*_*s*_ = 11.96) using conventional photolithography and thermal evaporation processes. The bar structures made from 200 nm-thick aluminum in the bottom layer were first patterned on the bare Si substrate, followed by 10 μm polyimide spacer (*ε*_*p*_ = 2.96 + 0.27*i*) on it. After the cure heating cycles, the four-SRR structures in the middle layer were aligned and patterned on the polyimide spacer. Finally, the second 10 μm polyimide spacer was applied followed by the patterning process of the I-shaped structures. The entire area of the EIA sample is 15 × 15 mm^2^.

### Measurement

The amplitude transmission of the EIA metamaterial was measured using the photoconductive antenna based *8f* confocal transmission (THz-TDS) system with a minimum beam diameter illuminated on the sample being 3.5 mm, while the amplitude reflection spectrum was measured using a same-type but reflective THz-TDS system. The amplitude transmission and reflection are defined as|*t*(*ω*)| = |*E*_st_(*ω*)/*E*_i_(*ω*)| and|*r*(*ω*)| = |*E*_sr_(*ω*) / *E*_i_(*ω*)|, respectively; where *E*_st_(*ω*) is measured transmission spectrum, *E*_sr_(*ω*) is the reflection spectrum. They are both normalized by *E*_i_(*ω*) with respect to the transmission or reflection amplitude of air and an aluminum coated silicon wafer, respectively.

### Simulations

All the simulated results were obtained by using finite-difference-time-domain (FDTD) method in the *CST Microwave Studio*. The amplitude transmission (|S_21_|) and reflection (|S_11_|) spectra were calculated using waveguide port excitations of a single unit cell. Perfect magnetic and perfect electric boundary conditions were applied along the *y* and *x*, or *x* and *y* directions to realize the *y*-polarized or *x*-polarized incidence, respectively. Open boundary conditions were applied on the *z* direction with vacuum of certain thickness being the spacer on both the resonator and substrate sides. The current distributions in Fig. 4 are plotted by setting H-field/Surface current monitor at peak absorption frequency.

## Additional Information

**How to cite this article**: Zhang, X. *et al*. Electromagnetically induced absorption in a three-resonator metasurface system. *Sci. Rep*. **5**, 10737; doi: 10.1038/srep10737 (2015).
